# MPGH-FS: A Hybrid Feature Selection Framework for Robust Multi-Temporal OBIA Classification

**DOI:** 10.3390/s25185933

**Published:** 2025-09-22

**Authors:** Xiangchao Xu, Huijiao Qiao, Zhenfan Xu, Shuya Hu

**Affiliations:** 1 College of Geological and Surveying Engineering, Taiyuan University of Technology, Taiyuan 030024, China; 2023510724@link.tyut.edu.cn (X.X.); 2024521143@link.tyut.edu.cn (Z.X.); 2024510753@link.tyut.edu.cn (S.H.); 2 Taiyuan Research Institute of China Coal Technology & Engineering Group, Taiyuan 030006, China

**Keywords:** feature selection, MICC, GA, HC, OBIA

## Abstract

Object-Based Image Analysis (OBIA) generates high-dimensional features that frequently induce the curse of dimensionality, impairing classification efficiency and generalizability in high-resolution remote sensing images. To address these challenges while simultaneously overcoming the limitations of single-criterion feature selection and enhancing temporal adaptability, we propose a novel feature selection framework named Mutual information Pre-filtering and Genetic-Hill climbing hybrid Feature Selection (MPGH-FS), which integrates Mutual Information Correlation Coefficient (MICC) pre-filtering, Genetic Algorithm (GA) global search, and Hill Climbing (HC) local optimization. Experiments based on multi-temporal GF-2 imagery from 2018 to 2023 demonstrated that MPGH-FS could reduce the feature dimension from 232 to 9, and it achieved the highest Overall Accuracy (OA) of 85.55% and a Kappa coefficient of 0.75 in full-scene classification, with training and inference times limited to 6 s and 1 min, respectively. Cross-temporal transfer experiments further validated the method’s robustness to inter-annual variation within the same area, with classification accuracy fluctuations remaining below 4% across different years, outperforming comparative methods. These results confirm that MPGH-FS offers significant advantages in feature compression, classification performance, and temporal adaptability, providing a robust technical foundation for efficient and accurate multi-temporal remote sensing classification.

## 1. Introduction

High-spatial-resolution satellite imagery (e.g., WorldView, Gaofen) has emerged as a vital data source for urban management and ecological monitoring, owing to its ability to capture fine geometric structures and surface textures [1]. However, while enhanced spatial resolution improves the characterization of land surface features, it simultaneously leads to greater spectral or textural heterogeneity within homogeneous land cover categories. This poses considerable challenges for conventional pixel-based classification methods, which typically rely solely on the spectral characteristics of individual pixels without accounting for spatial context. Consequently, such methods often fail to delineate semantically meaningful land objects and are prone to generating salt-and-pepper noise in the classification results, ultimately reducing both accuracy and spatial coherence [2,3].

Object-Based Image Analysis (OBIA) has become an effective strategy to address the complexity of high-resolution remote sensing imagery. By performing multi-scale segmentation to generate homogeneous image objects, OBIA integrates multi-source information, including spectral, texture, geometric, and spatial contextual data, to effectively enhance the semantic consistency and structural integrity of land cover classification [4]. While OBIA has emerged as a mainstream approach for high-resolution remote sensing interpretation [2,5], it also introduces several critical challenges. The explosion in feature dimensionality associated with image objects can lead to the well-known curse of dimensionality, where the computational and storage overhead of redundant features significantly reduces processing efficiency. Furthermore, the presence of irrelevant or weakly discriminative features may degrade classification accuracy [6].

To address the performance bottlenecks induced by high-dimensional feature spaces and inherent redundancy, feature selection has been widely recognized as a critical strategy for improving classification efficiency and accuracy. By eliminating redundant and noisy features from the original high-dimensional feature pool, feature selection effectively reduces model complexity and mitigates the risk of overfitting [7,8].

Existing feature selection methods can be categorized into three types based on their search strategies: optimal, heuristic, and randomized [9]. The optimal search strategy aims to identify the theoretically optimal feature subset by systematically evaluating all possible combinations, such as in exhaustive enumeration or the branch-and-bound algorithm [10]. Although these methods ensure global optimality due to their mathematical rigor, the combinatorial explosion associated with high-dimensional data renders them computationally infeasible. For instance, for data containing N features, the theoretical search space consists of $2^{N}\;-\;1$ combinations, making the optimal search strategy impractical for high-dimensional remote sensing applications. The heuristic search strategy employs a greedy algorithm approach to construct subsets through feature ranking and stepwise selection. Its core process includes quantifying feature importance based on evaluation criteria (such as Relief-F, mutual information, etc.) and iteratively optimizing the subset through forward selection or backward elimination. For example, Yu et al. [11] used both Relief-F and Max–Min-Associated (MNA) methods to evaluate the contribution of 63 features to global land cover classification, selecting feature subsets by progressively including the top-ranked features. Hossain et al. [12] applied Normalized Mutual Information (NMI) to quantify the correlation between hyperspectral image bands and target classes, and reduced dimensionality by iteratively discarding the least relevant bands. Despite its computational efficiency [13], this strategy remains fundamentally limited as it prioritizes feature–class relevance while neglecting inter-feature redundancy, which may result in suboptimal subset selection and reduced classification performance [6,14]. The randomized search strategy typically utilizes swarm intelligence optimization algorithms, which simulate biological cooperative behaviors to perform random searches in the solution space and achieve optimization in a distributed manner [15]. Representative methods include Genetic Algorithm (GA) and Particle Swarm Optimization (PSO). Siedlecki and Sklansky [16,17] were the first to apply GA for feature selection and subsequently validated its efficiency and applicability in feature selection problems. Agrafiotis and Cedeño [18] adapted the original PSO, which was designed for continuous space optimization problems, to feature selection by using binary encoding. To enhance the optimization capability of the original algorithm, Wang et al. [19] introduced the Lévy Flight (LF) strategy into the Ant Lion Optimizer (ALO), achieving both band reduction and improved classification accuracy in hyperspectral image feature selection. Compared to optimal and heuristic searches, these swarm optimization algorithms yield higher accuracy while offering a favorable balance between effectiveness and computational efficiency through probabilistic optimization in the solution space. However, their performance remains sensitive to several factors such as algorithm complexity, initial parameter settings, and the coupling mechanism with classifiers, which may result in variable accuracy–efficiency performance [20]. Overall, the three types of feature search strategies exhibit distinct strengths and limitations in terms of optimization capability and computational efficiency. Optimal search methods guarantee theoretically optimal solutions but incur high computational costs. In the contrast, heuristic approaches are highly efficient but tend to be limited in its optimization ability. To bridge the gap between these two extremes, randomized search strikes a balance between accuracy and computational efficiency.

Given the complementary nature of these strategies, researchers have gradually explored the integration of multiple methods to balance search quality with computational cost. One approach focuses on intra-strategy fusion, which enhances selection accuracy by integrating different techniques within a single search strategy. For example, Guha et al. [21] proposed a heuristic search method called Mutually Informed Correlation Coefficient (MICC), which integrates Mutual Information (MI) and the Pearson Correlation Coefficient (PCC) to reduce feature redundancy. Similarly, Mafarja and Mirjalili [22] used the Simulated Annealing (SA) algorithm to enhance the best feature set obtained by the Whale Optimization Algorithm (WOA). As a further development, Wang et al. [23] successfully applied this strategy to cross-scene hyperspectral image band selection tasks, improving classification accuracy by approximately 5% compared to traditional methods on the Indiana and Pavia cross-scene hyperspectral datasets. Another line of research involves cross-strategy fusion, where hybrid frameworks combine distinct search mechanisms to balance efficiency and optimization capability. For instance, Ghosh et al. [24] proposed a two-stage feature selection model that integrates Relief-F heuristic search with GA randomized search for efficiently identifying cancer-related genes in microarray data. Xie et al. [25] employed Fuzzy C-Means (FCM) clustering to filter out redundant bands in hyperspectral imagery, followed by iterative optimization using the Grey Wolf Optimizer (GWO). Although hybrid feature selection methods have made notable progress in addressing high-dimensionality, most existing approaches are designed for single-temporal imagery analysis and fail to accommodate temporal variability. As a result, when applied to multi-temporal remote sensing tasks, these methods must be executed repeatedly for each image, leading to substantial redundancy, increased computational burden, and greater inconsistency in classification results. Therefore, developing a feature selection framework that balances feature compression efficiency with temporal robustness has become a critical scientific challenge for enhancing cross-temporal classification performance of high-resolution remote sensing imagery.

To address the above challenge, this study proposes a novel feature selection method named MPGH-FS, which integrates heuristic search-based preselection with randomized search. The method first constructs a dual-criterion filtering mechanism based on MI and PCC to effectively reduce the feature space. It then combines the global search capability of GA with the local optimization ability of the Hill Climbing (HC) algorithm, forming a hybrid optimization framework to enhance solution quality and convergence efficiency. The proposed method is evaluated using Gaofen-2 satellite imagery from five temporal phases (2018, 2019, 2020, 2022, and 2023; owing to the absence of imaging passes in 2021). The evaluation focuses on feature selection efficiency, as well as the cross-temporal transferability and stability of the resulting feature subsets. These findings can contribute methodological insights for feature compression and multi-temporal model transfer in object-based classification and, to some extent, support temporal remote sensing applications such as urban dynamics monitoring.

## 2. Study Area and Data

**Figure 1:**
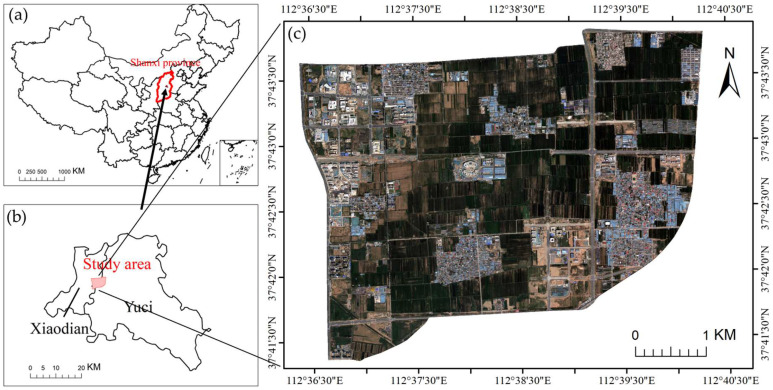
Maps showing the geographical context and remote sensing imagery of the study area: (**a**) the national position of Shanxi province; (**b**) the specific location of the study area; (**c**) a GF-2 image acquired in July 2020.

The study area is the Technology Innovation City, part of the Shanxi Transformation Comprehensive Reform Demonstration Zone (37°44′ N–37°47′ N, 112°33′ E–112°38′ E), situated in the southern region of Taiyuan City, Shanxi Province, China. Geographically, it extends from Wuluo Street in the north to Fendong Street in the south, and from Malianying Road in the west to the Taiyuan–Zhongwei–Yinchuan railway (Taiyu region) in the east, covering an area of approximately 20 km^2^ (Figure 1). As the first national-level comprehensive reform demonstration zone initiated at the provincial level and promoted systematically, the area was officially approved by the State Council in 2017. Strategically positioned as both a hub for technological innovation and a nucleus for emerging industries in central and western China, the zone focuses on the development of strategic sectors such as new energy, new materials, intelligent manufacturing, and information technology.

The study area was originally a typical suburban agricultural region, with land use dominated by villages and farmland. Since the official approval of the demonstration zone in 2017, the area has undergone rapid urbanization driven by national policies. Construction land has expanded continuously, while agricultural land and village spaces have been extensively transformed. As a result, surface cover types have become increasingly complex and diverse, and the area has gradually evolved into a new urban functional zone cantered on industrial parks, research and development bases, and supporting infrastructure. This area was selected as the study site not only because it exhibits typical characteristics for remote sensing classification, such as rapid land use change and complex land cover types, but also because its rapidly urbanizing landscape provides an ideal experimental setting for evaluating the temporal adaptability of feature selection methods for high-resolution remote sensing imagery.

**Figure 2:**
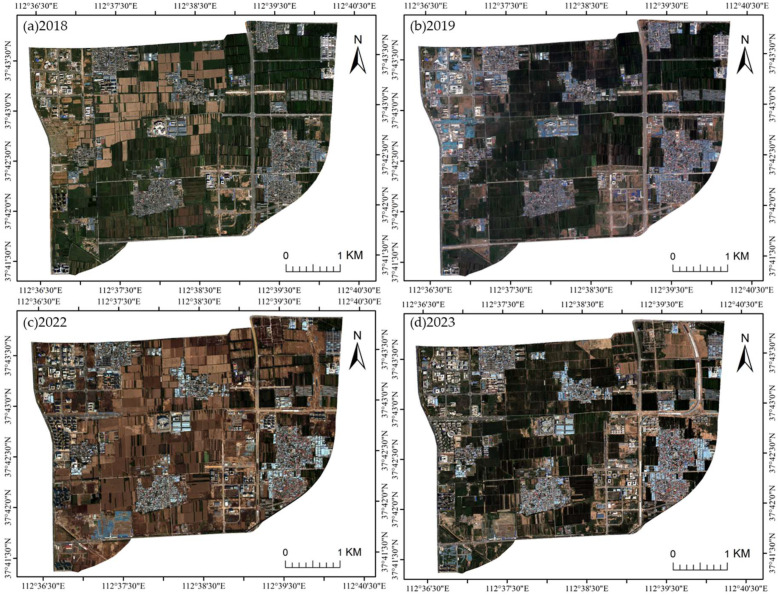
Multi-temporal GF-2 images used for cross-year transfer testing.

This study used remote sensing imagery acquired by the Gaofen-2 (GF-2) satellite of the China High-resolution Earth Observation System (CHEOS) as the primary data source. Five temporal scenes acquired in September 2018, July 2019, July 2020, October 2022, and July 2023 were selected. Among them, the July 2020 image (Figure 1c) served as the primary experimental data for feature selection, aiming to extract a representative optimal feature subset. The remaining four images (Figure 2) were used as transfer test data to evaluate the transferability and stability of each feature subset across multi-temporal imagery, thereby verifying the proposed feature selection method’s adaptability and generalization performance over time.

## 3. Method

**Figure 3:**
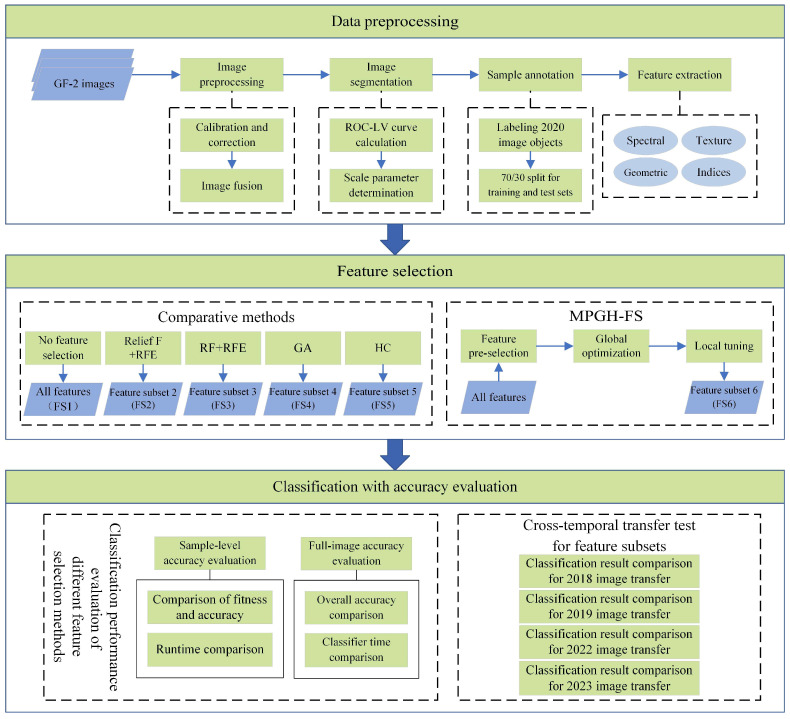
The overall framework of this study.

This study adopted a systematic three-stage processing framework (Figure 3): First, three GF-2 images were subjected to radiometric correction and data fusion, followed by multiscale segmentation to construct semantically consistent image objects and extract spectral, textural, geometric, and index features. Next, a hierarchical feature selection method, MPGH-FS, was introduced. It integrates statistical correlation-based pre-filtering, global search using GA, and local refinement with HC to achieve collaborative optimization. Finally, comparative experiments were conducted using the full feature set and the feature subsets selected by Relief F + Recursive Feature Elimination (RFE), Random Forest (RF) + RFE, GA, HC, and MPGH-FS to evaluate classification accuracy. In addition, cross-temporal transfer experiments were performed to verify the temporal robustness of each selected feature subset.

### 3.1. Image Segmentation

To construct semantically consistent image objects, this study employed an object-based multi-scale segmentation method to process remote sensing imagery. The Multi-Resolution Segmentation (MRS) algorithm on the eCognition Developer V9.5 platform was used to perform object-level segmentation of the image.

The segmentation results are primarily influenced by three parameters: (1) the Scale parameter, which determines the granularity of segmented objects and plays a critical role in segmentation quality; (2) the Shape parameter, which balances the influence of spectral features and shape features during segmentation; and (3) the Compactness parameter, which regulates the compactness of the segmented objects. Among these, the scale parameter is crucial to avoid both “over-segmentation” and “under-segmentation” phenomena. Improper setting of this parameter can severely disrupt the subsequent classification accuracy. To determine the optimal segmentation scale, this study employed Local Variance (LV) and the Rate Of Change of Local Variance (ROC-LV) as reference indicators. LV quantifies the spectral heterogeneity within image objects, where higher values reflect greater internal variability [26]. When the segmentation scale approaches the natural scale of ground objects, the ROC-LV curve typically exhibits prominent peaks [27,28], which often correspond to the scale thresholds of typical object boundaries in the image. The scale–ROC-LV curve generated using the ESP II (estimation of scale parameter) plugin enables the objective identification of optimal segmentation scale parameters for different land cover types. The calculation formula is as follows:

(1) $$LV\;=\frac{1}{M}\sum_{i=1}^{M}\sigma_{i}$$

(2) $$ROC=\frac{LV-{LV}_{-}}{{LV}_{-}}$$

Here, $\sigma_{i}$ represents the standard deviation of pixel values within the i-th segmented object at a given segmentation scale, and M denotes the total number of segmented objects in the entire image. LV is the local variance at the current segmentation scale, while ${LV}_{-}$ refers to the local variance at the previous, smaller segmentation scale. Specially, for each segmented object, the standard deviation of its internal pixel values is first calculated. Then, the average of these standard deviations across all objects in the image is computed to derive a local variance value for that particular scale. To identify scale-sensitive changes, the rate of change of local variance is calculated by analyzing the curvature of local variance values across different scale parameters.

### 3.2. Feature Extraction

To comprehensively represent the multi-dimensional information of image objects, a total of 232 variables (Table 1) were extracted, covering four major categories: spectral features, geometric features, texture features, and index features. These variables were used in the subsequent feature selection process.

Among these, spectral features include object brightness, mean and standard deviation of each band, maximum difference, and hue, intensity, and saturation, resulting in a total of 13 features. These features primarily reflect the reflectance characteristics and color distribution patterns of image objects in different spectral channels. Geometric features describe the spatial morphology of the objects, including 8 attributes such as area, boundary length, major axis length, width, aspect ratio, and pixel count, which express the geometric structure and shape differences of the objects. Index features include common visible light indices and vegetation indices (such as NDVI, ExG, VARI, MGRVI, RGBVI, etc.), with a total of 11 features, primarily used to enhance the differentiation between different land cover types. Texture features, which are particularly valuable for distinguishing built-up areas and heterogeneous surfaces, are derived from eight statistical metrics of the Gray-Level Co-occurrence Matrix (GLCM), including contrast, entropy, and homogeneity. By computing each metric across multiple bands and four orientations (0°, 45°, 90°, and 135°), a total of 200 texture variables were generated, effectively capturing the internal spatial arrangement and structural patterns of image objects. Compared to other types of features, texture features exhibit significantly higher dimensions, primarily due to the feature expansion resulting from the multi-directional, multi-band, and multi-property cross-combinations. Although such high dimensionality may introduce some redundancy risks, texture information plays an irreplaceable role in the recognition of targets such as buildings in urban remote sensing applications [29]. Urban buildings typically present regular geometric shapes and well-defined structural boundaries, displaying typical characteristics of high contrast and strong directionality in the texture domain [30]. Therefore, texture features play a key role in enhancing the accuracy of urban building target recognition. To ensure consistency and comparability across all feature types, feature extraction was performed under a unified segmentation scale, providing a standardized basis for subsequent feature selection and classification tasks.

### 3.3. MPGH-FS Algorithm

**Figure 4:**
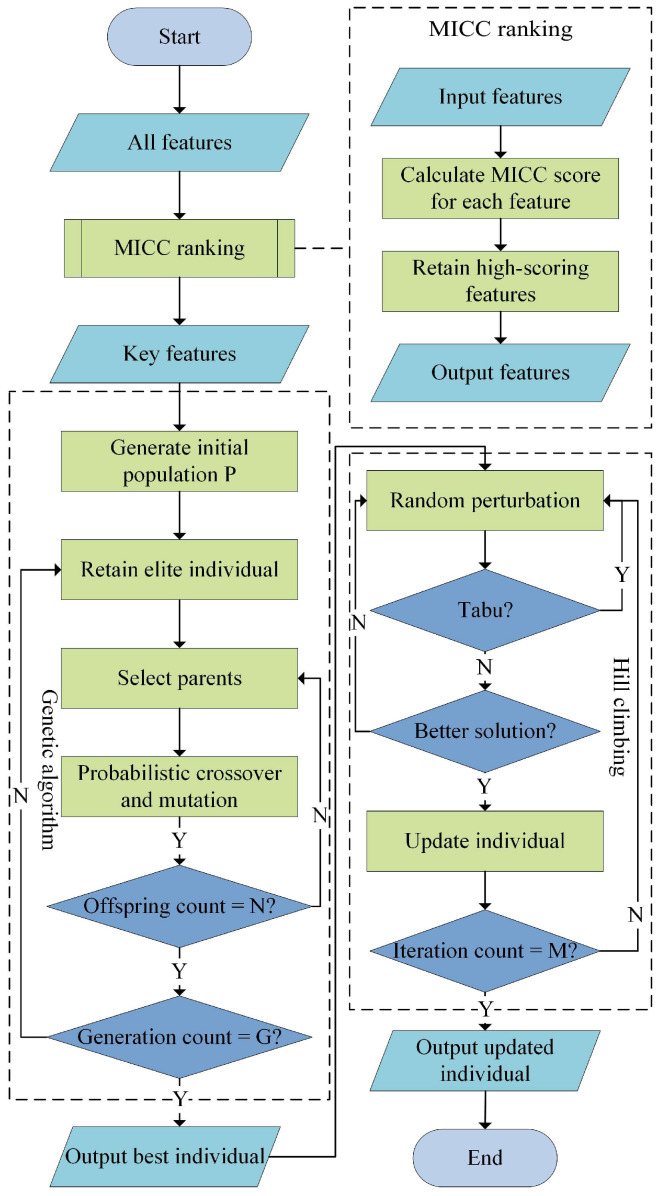
Flowchart of the proposed MPGH-FS algorithm.

To improve the efficiency and accuracy of feature selection, this study proposes a method called MPGH-FS, which integrates MI and PCC-based filtering (MICC), GA, and HC. As illustrated in Figure 4, the MPGH-FS framework consists of three main stages: (1) MICC pre-filtering: Features are filtered based on mutual information and Pearson correlation coefficient to reduce the search space; (2) Global search via GA: The GA performs population evolution within the reduced feature space to explore globally optimal feature subsets; (3) Local optimization via HC: The optimal solution produced by the GA is further refined through neighborhood perturbation to accelerate convergence.

#### 3.3.1. Preliminary Feature Filtering Using MICC

In the feature selection framework of this study, the MICC method was employed as an initial filtering tool for extracted features, primarily to evaluate and rank their discriminative potential. This method, originally proposed by Guha, Ghosh, Bhowmik and Sarkar [21], constructs a unified evaluation metric for feature filtering by MI and PCC. Specifically, MI measures the relevance between each feature and the class labels, reflecting its discriminative power, while PCC assesses the linear correlation among features to identify and eliminate redundancy. Through the combination of MI and PCC, MICC can effectively filter out features with weak discriminative ability and high redundancy, thereby providing a more concise search space for subsequent feature selection.

The MI between a feature $F_{i}$ and the class labels Y is mathematically defined as

(3) $$R_{mi}\left(F_{i},Y\right)=\sum_{f\in F_{i}}\sum_{y\in Y}P_{\left(F_{i},Y\right)}\left(f,y\right)\log\left(\frac{P_{\left(F_{i},Y\right)}\left(f,y\right)}{P_{F_{i}}\left(f\right)P_{Y}\left(y\right)}\right)$$

Here, $F_{i}$ denotes the range of values for the i-th feature, and Y represents the range of the class variable. $P_{\left(F_{i},Y\right)}\left(f,y\right)$ is the joint probability distribution of $F_{i}$ and Y, while $P_{F_{i}}\left(f\right)$ and $P_{Y}\left(y\right)$ are the marginal probability distributions of $F_{i}$ and Y, respectively. A higher mutual information value indicates a stronger association between the feature and the class labels.

The redundancy between features is measured using PCC:

(4) $$R_{pcc\left(F_{i},F_{k}\right)}\;=\;\frac{\sum_{j=1}^{n}\left(F_{i}^{\left(j\right)}-{\bar{F}}_{i}\right)\left(F_{k}^{\left(j\right)}-{\bar{F}}_{k}\right)}{\sqrt{\sum_{j=1}^{n}{\left(F_{i}^{\left(j\right)}-{\bar{F}}_{i}\right)}^{2}}\sqrt{\sum_{j=1}^{n}{\left(F_{k}^{\left(j\right)}-{\bar{F}}_{k}\right)}^{2}}}$$

Here, $F_{i}^{\left(j\right)}$ and $F_{k}^{\left(j\right)}$ represent the values of the j-th sample for features $F_{i}$ and $F_{k}$, respectively. ${\bar{F}}_{i}$ and ${\bar{F}}_{k}$ denote the mean values of features $F_{i}$ and $F_{k}$ across all samples, and n is the number of samples. A higher correlation coefficient between two features indicates a higher degree of redundancy.

A weighted fusion strategy is adopted to construct the overall evaluation function for feature $F_{i}$:

(5) $$\mathrm{SCORE}\left(F_{i}\right)=w_{1}R_{\mathrm{mi}}\left(F_{i},Y\right)-w_{2}\left(\frac{\sum_{k=1}^{m}R_{\mathrm{pcc}}\left(F_{i},F_{k}\right)}{m}\right)$$

Here, m is the total number of features, and $w_{1}$ and $w_{2}$ are user-defined weighting coefficients that satisfy $w_{1}+w_{2}=1$, used to balance the final score between feature relevance and redundancy. In this study, to ensure that MICC emphasizes the relevance between features and class labels, $w_{1}$ was set to 0.9.

#### 3.3.2. GA and HC Search Strategies

To efficiently search in high-dimensional feature spaces, MPGH-FS combined global exploration via genetic algorithms and local optimization via hill climbing.

GA is a typical swarm intelligence optimization method with strong global search capabilities, capable of discovering potential optimal solutions in complex solution spaces [31]. As the core optimization component of MPGH-FS, GA performs a sequence of operations including population initialization, fitness evaluation, selection, crossover, and mutation. During the selection phase, a combination of tournament selection and elitist preservation strategies is employed to guide the search toward high-quality solutions. The crossover operation recombines parental information through single-point crossover to enhance population diversity. Finally, the mutation operation randomly flips gene positions in individuals to avoid local optima and improve global exploration. The synergy of these three operations enables GA to effectively explore potentially optimal feature subsets in high-dimensional spaces and provides a high-quality initial solution set for subsequent local optimization. In contrast to the global search strategy of GA, HC is a greedy local search method that efficiently finds better solutions in the neighborhood of an initial solution [32]. In the MPGH-FS framework, HC is applied to the elite individual obtained from GA, aiming to refine solution quality and accelerate convergence. The local search proceeds by generating neighboring candidates around the current solution, evaluating them using a fitness function specifically designed for classification tasks, and iteratively updating the solution when an improvement is detected. This process continues until a predefined termination criterion is met. By leveraging the high-quality starting point provided by GA, HC enhances the ability to locally refine solutions of the model while maintaining low computational cost, ultimately improving classification performance under conditions of good initialization.

In summary, GA enables global exploration of the solution space, while HC performs further local refinement. The complementary collaboration between the two ensures that MPGH-FS achieves both search efficiency and classification performance in feature selection tasks.

#### 3.3.3. Fitness Function

To fit the concept of individuals in swarm intelligence algorithms such as GA, binary encoding is commonly used to formally represent the candidate feature set [33]. Let the original feature space have a dimensionality of m; then, each individual can be represented as a binary vector of length m:

(6) $$X\;=\;\left(x_{1},x_{2},\ldots ,x_{m}\right),\;\;\;\;x_{i}\in \left\{0,1\right\}$$

where $x_{i}\;=\;1$ indicates that the i-th feature is selected, and $x_{i}\;=\;0$ indicates that the feature is not selected.

To evaluate the quality of a candidate solution, the fitness function is defined as follows [34]:

(7) $$fitness\left(X\right)=\frac{Accuracy\left(X\right)}{1+\lambda {\left\|X\right\|}_{0}}$$

where $Accuracy\left(X\right)$ denotes the classification accuracy based on the selected feature subset X, and ${\left\|X\right\|}_{0}\;=\;\sum_{i=1}^{m}x_{i}$ represents the number of selected features. The parameter λ is a regularization coefficient that controls the influence of feature subset size on the fitness value. Based on preliminary experiments, λ is set to 0.008 in this study.

#### 3.3.4. MPGH-FS Algorithm Procedure

The detailed procedure of the MPGH-FS algorithm is shown in Algorithm 1. First, all features in the experimental dataset were ranked based on their MICC scores, and the bottom pct=50% of features were removed to reduce the search space.

**Algorithm 1.** The proposed MPGH-FS**Input:** 2020 sample dataset containing all features; *pct*: percentage of lowest-ranked features to discard; *P*: population size for GA; *G*: number of generations for GA; *M*: maximum number of iterations for HC; *pc*: crossover probability; *pm*: mutation probability.
**Output:** *fos*: Final optimal feature subset; *ff*: fitness value of *fos*.
1. **For** each feature in dataset, compute its MICC score using Equation (5);
2. Sort features by MICC score in descending order;
3. Dataset with the lowest-ranked pct proportion of features removed → *selected_data*;
4. Genetic algorithm phase:
    4.1 Initialize a population *pop* of size *P* with random individuals;
    4.2 **For** *generation* = 1 to G, **do:**
            4.2.1 Evaluate the fitness value of each individual in *pop*;
            4.2.2 Identify the best individual in *pop* → *bi*;
            4.2.3 Store *bi* in the first position of the new population: *new_pop*[0] = *bi*;
            4.2.4 **For** *i* = 1 to P − 1, **do:**                    (1) Randomly select 5 individuals *idv[0–4]* from *pop*;
                    (2) Evaluate their fitness values: fitness(*idv[0–4]*);
                    (3) Select the individual with the highest fitness value: *idv[h]*;
                    (4) Assign it to *parent1* as the selected parent individual;
                    (5) **Repeat** (1)–(4) to select *parent2*;
                    (6) **If** random() < *pc*, perform crossover between *parent1* and *parent2* → *offspring*;                                     **Else**, *parent1* → *offspring*;
                    (7) **If** random() < *pm*, mutate *offspring* by flipping one random bit;
                    (8) **Add** *offspring* to *new_pop*[*i*];
            4.2.5 Replace *pop* with *new_pop*;
5. Hill climbing phase:
    5.1 **Set** current individual *ci* = *bi*;
    5.2 Initialize an empty tabu list *T* = ∅;
    5.3 **For** *i* = 0 to *M* − 1, **do:**            5.3.1 Randomly flip one or two bits in the current individual *ci*;
            5.3.2 **Repeat:**
                       Generate a new individual *ni* by applying the selected perturbation to *ci*;
                    **Until** *ni* ∉ *T*;
            5.3.3 **Add** *ni* to *T*;
            5.3.4 **If** fitness(*ni*) > fitness(*ci*):
                       **Set** *ci* ← *ni*;
6. *ff* = fitness(*ci*), *fos* = *ci*;
7. **Return** *fos* and *ff*.

After MICC pre-filtering, GA was applied to perform a global search for the optimal feature subset. The GA’s core operations include elitism, tournament selection, single-point crossover, and bit-flip mutation. To determine the optimal hyperparameters, a series of univariate experiments based on the coordinate descent idea were conducted, including population size (50–200), crossover probability (0.1–0.9), and mutation probability (0.1–0.9). The final settings were ultimately determined as follows: population size P=90, crossover probability pc=0.7, and mutation probability pm=0.7. After G=60 generations, the historically best individual was output as the optimal solution. During algorithm execution, the initial population was randomly generated, with each individual represented by a binary string of length equal to the feature dimensionality. In each generation, the fitness evaluation and an elitism mechanism were used to preserve the current best solution. The remaining individuals were generated via 5-way tournament selection, single-point crossover, and bit-flip mutation to form the next generation. Iterations continued until the maximum number of generations is reached. In the final stage, the improved HC algorithm was applied to locally refine the best individual produced by GA. Considering the binary encoding nature of the feature selection problem, a random perturbation strategy was designed to flip one or two bits, effectively adding or removing features. To prevent redundant evaluations and accelerate convergence, a short-term memory mechanism inspired by tabu search was introduced. A dynamic tabu list with a capacity of 500 was maintained to avoid redundant evaluations, significantly improving search efficiency. A maximum of M=10,000 iterations was set in this stage to ensure a balance between local search depth and computational cost.

## 4. Results and Analysis

### 4.1. Image Segmentation Results

**Figure 5:**
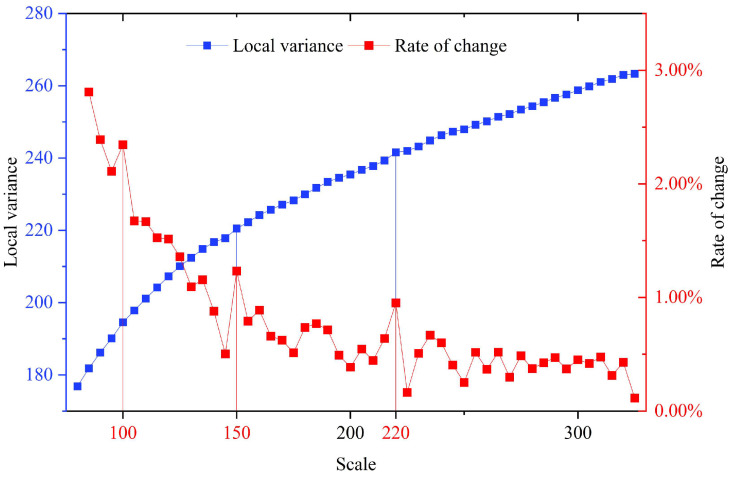
ROC-LV curve for scale parameter evaluation.

The optimal segmentation scale parameter was evaluated through a combined approach integrating the ROC-LV method and visual interpretation, yielding both quantitative and qualitative results. Based on the spatial characteristics of typical land-cover objects and preliminary experiments, the search range for the scale parameter was set from 80 to 325 with an increment of 5. A starting scale of 80 facilitated the detection of fine-grained objects (e.g., water bodies and small buildings), while a maximum scale of 325 encompassed large structures and agricultural patches. To ensure that the segmentation objects maintain regular shapes and closely resemble actual object boundaries, the shape and compactness parameters were fixed at 0.7 and 0.6, respectively. Based on the predefined parameter settings, segmentation experiments were systematically carried out, during which the LV and its rate of change across multiple scale levels were computed to construct an ROC-LV curve for optimal scale determination (Figure 5).

**Figure 6:**
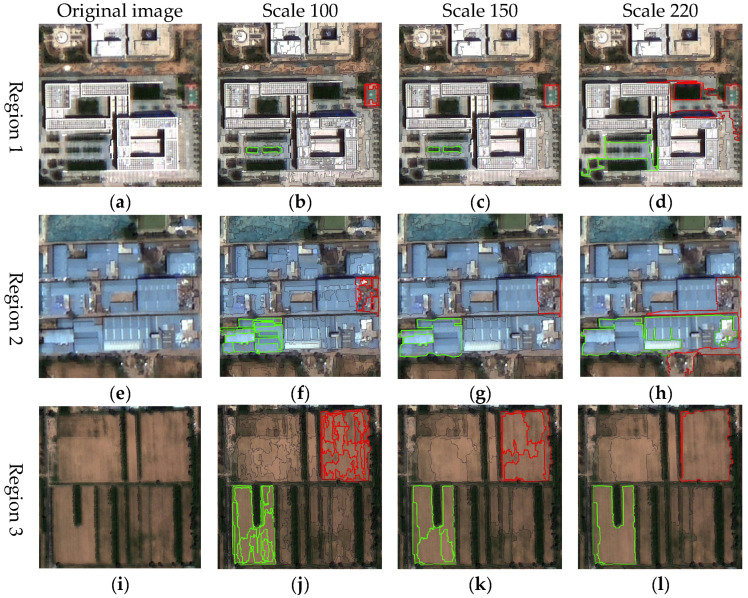
Comparison of local segmentation results at candidate scales: (**a**,**e**,**i**) Original images; (**b**,**f**,**j**; **c**,**g**,**k**; **d**,**h**,**l**) Segmentation results at scales 100, 150, and 220, respectively, with red and green boxes highlighting representative regions for visual assessment.

In Figure 5, the blue and red curves represent the LV values (left *y*-axis) and ROC-LV values (right *y*-axis), respectively. The results demonstrated that as the segmentation scale increases, the LV exhibits an overall upward trend, while its rate of change gradually declines and eventually stabilizes. Notable fluctuations in the ROC-LV curve were observed at scales of 100, 150, and 220, where prominent peaks emerge. These peaks suggested that, at these scales, the internal grayscale variation, texture heterogeneity, and boundary clarity of land-cover objects were maximized. This indicated the segmentation effectively captures object structures across different spatial granularities. To assess the segmentation performance of the peak scale parameters identified from the ROC-LV curve, three representative sub-regions were selected for visual assessment. Segmentation results using candidate parameters of 100, 150, and 220 are presented in Figure 6.

In region 1, a segmentation scale of 100 results in obvious over-segmentation: the playground area outlined in red (Figure 6b) was clearly delineated but internally fragmented into numerous sub-objects, compromising spatial integrity. At scale 150 (Figure 6c), the playground was accurately extracted with a clear and complete boundary, yielding the best segmentation performance. In contrast, scale 220 (Figure 6d) led to under-segmentation, merging the playground with surrounding buildings into a single large object with unclear boundaries. Region 2 exhibited similar trend. At scale 100 (Figure 6f), the debris area (upper-right red box) and the factory area (lower-left green box) showed clear signs of over-segmentation, with objects fragmented into multiple parts. At scale 220 (Figure 6h), under-segmentation led to blurred boundaries and fusion with nearby roads. The segmentation at scale 150 (Figure 6g) achieved the best overall balance, maintaining clear object boundaries and preserving the integrity of major structures, despite minor internal refinement. In region 3 (Figure 6i–l), primarily consisting of extensive bare land patches, a scale of 220 better preserved the overall integrity, generating complete and coherent objects (Figure 6l). In comparison, a scale of 150 (Figure 6k) maintained boundary clarity but divided the bare land into multiple sub-objects, slightly diminishing spatial continuity. Over-segmentation was most severe at scale 100 (Figure 6j), with extensive fragmentation of the bare land.

By synthesizing the segmentation results across the three regions, it was evident that a scale of 100 generally led to over-segmentation, resulting in significant object fragmentation. Although a scale of 220 effectively maintains the overall integrity of large homogeneous areas such as bare land, it tended to cause under-segmentation in most other land cover types, leading to blurred boundaries and merged objects. In contrast, a scale of 150 achieved a balance between boundary accuracy and structural coherence, delivering consistently good segmentation performance across different land cover types. Therefore, the scale of 150 was adopted in this study as the unified segmentation parameter for subsequent object-based feature extraction and classification analysis.

### 4.2. Feature Selection Results and Analysis

This study used the GF-2 image from July 2020 as the experimental dataset for feature selection. A total of 1079 valid samples were manually labelled, including 732 construction land, 196 bare land, 133 vegetation, and 18 water body samples. A stratified random sampling method was adopted to split the dataset into a training set and a test set in a 7:3 ratio, ensuring that the class distribution remained consistent across both sets. The training set was used for feature subset selection, while the test set served for accuracy validation. To evaluate the effectiveness of the proposed MPGH-FS method, six experimental schemes based on different feature selection strategies were designed. For clarity, a summary of the compared methods and their corresponding descriptions is provided in Table 2.

These methods include no feature selection, Relief-F + RFE, RF + RFE, standard GA, standard HC, and the proposed MPGH-FS method. No Feature Selection served as the baseline, retaining all 232 original features to provide a reference for comparison. Relief-F combined with RFE was selected as a benchmark method, representing a classical feature selection paradigm that integrates statistical evaluation with sequential search. In this approach, the Relief-F algorithm first evaluates feature importance by calculating the distance differences between each feature’s nearest hit (the closest neighbor within the same class) and nearest miss (the closest neighbor from a different class), subsequently ranking the features based on these criteria. Following this, RFE is employed to iteratively remove the least important features based on the ranking to obtain the optimal feature subset. This combined approach has been widely adopted in the remote sensing field [35,36]. In contrast to the statistical foundation of Relief-F, we also incorporate RF as a representative machine learning-based feature selection method. RF operates through the construction of multiple decision trees, evaluating features during node splitting by their ability to reduce impurity through data partitioning. By aggregating the impurity reductions across all trees, RF naturally provides a measure of feature importance [37]. The overall importance of each feature is then derived by averaging impurity reductions across all trees in the ensemble, providing a robust, intrinsic measure of feature relevance. This combined strategy is also widely utilized for feature selection and dimensionality reduction in remote sensing image analysis [37,38]. Together, these two algorithms offer a methodological contrast to the randomized search algorithms GA, HC, and MPGH-FS, thereby facilitating a comprehensive evaluation of feature selection performance from multiple perspectives. For simplicity, the feature subsets generated by the five methods are denoted as FS1 to FS6.

Since the effectiveness of feature selection cannot be directly verified by the number of selected features, the performance of the feature selection algorithms was evaluated based on the accuracy of the classification results of the test samples. To eliminate the impact of hyperparameter differences on classification results, a unified Support Vector Machine (SVM) classifier was employed across all experiments, using a radial basis function (RBF) as the kernel function and a fixed regularization parameter C=1.0. All experiments were conducted on a hardware platform configured with an Intel Core i9-9900 processor (3.1 GHz) and 32 GB of DDR4 memory. The algorithm implementation is based on Python 3.11, ensuring both reproducibility and comparability of the experimental results.

#### 4.2.1. Feature Selection Based on the Relief-F + RFE and RF + RFE Algorithm

**Figure 7:**
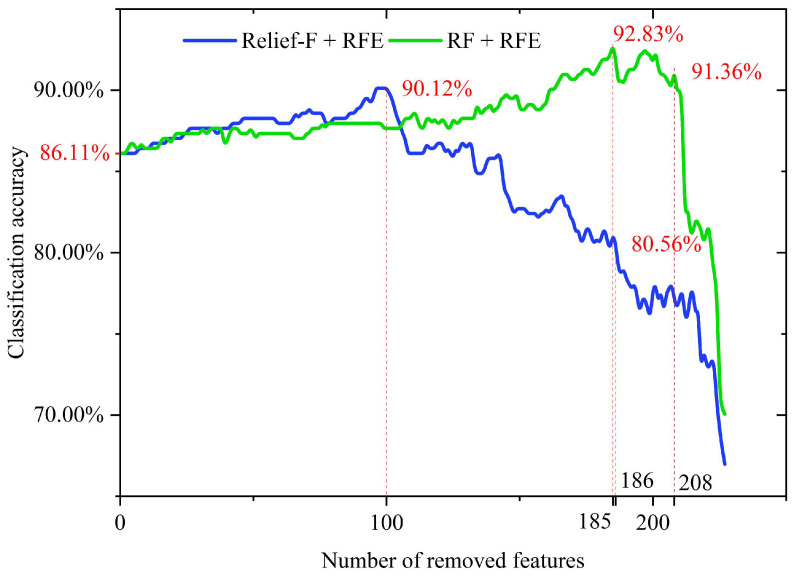
Classification Accuracy During Feature Removal by Relief-F + RFE and RF + RFE.

Figure 7 illustrates the performance curves of sample classification accuracy based on the Relief-F + RFE and RF + RFE algorithms. The horizontal axis represents the number of features removed (features are eliminated sequentially from lowest to highest according to their importance scores calculated by each algorithm), while the vertical axis donates the corresponding classification accuracy of the feature subsets with the SVM classifier.

As shown in Figure 7, the classification accuracy using all 232 features (FS1) is 86.11%. Starting from this baseline, the Relief-F + RFE algorithm iteratively removed low-importance features. As low-importance features were gradually eliminated, the classification accuracy steadily increased, reaching a peak of 90.12% after 100 features were removed, corresponding to 43% of original features. This indicated that Relief-F + RFE effectively identified noisy features or irrelevant features, enhancing the model’s discriminative capability. However, as the number of removed features increased, particularly beyond 186, representing 80% of the total, the classification accuracy rapidly decreased by about 6% from 80.56%, suggesting that high-importance features crucial for classification are being eliminated. The iteration curve of the RF + RFE algorithm showed a similar trend, but its accuracy peaks after 185 features were removed, reaching 92.83%, which was 2.71% higher than the maximum accuracy of Relief-F + RFE. This result demonstrated that the feature importance ranking based on RF is more effective, enabling higher classification performance with fewer retained features. However, once more than 208 features were removed, the classification accuracy sharply dropped by about 10% from 91.36%, indicating that the high-importance features identified by RF possess stronger discriminative power. Ultimately, based on the peak positions of the two accuracy curves, this study retained the top 132 features ranked by Relief-F to form feature subset FS2, and the top 47 features ranked by RF to form feature subset FS3.

#### 4.2.2. Feature Selection Based on Optimization Algorithms

**Figure 8:**
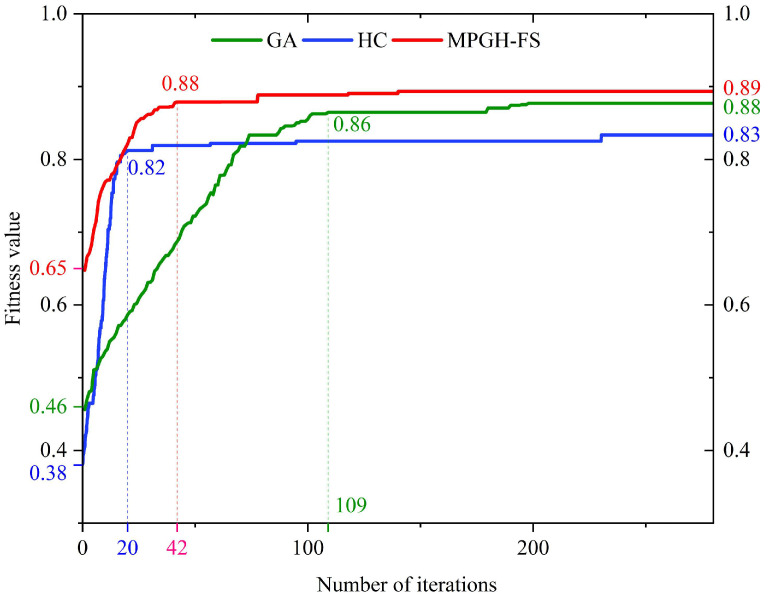
Fitness value evolution during algorithm iteration.

To evaluate the feature selection capabilities of three stochastic optimization algorithms, namely, the standard GA, HC, and the proposed MPGH-FS, each algorithm was independently executed 10 times. For a straightforward comparison of the convergence processes, the *x*-axis of the iteration curves was scaled to 280 generations. Figure 8 illustrates the evolution of fitness values across iterations for the three algorithms.

At the early stages of iteration, MPGH-FS demonstrated a significant advantage, achieving an initial fitness value of 0.65, which was approximately 0.19 and 0.27 higher than those of GA and HC, respectively. This indicated superior initial population quality achieved by incorporating the MICC filter-based feature selection strategy in MPGH-FS, which effectively removed noisy features and improves search efficiency.

During the subsequent evolutionary process, although the HC algorithm exhibited a rapid increase in fitness value in the early phase, it quickly stagnated and converged at a fitness value of 0.83, yielding the feature subset FS5. This suggested that while HC possessed certain local search capabilities, it lacked a mechanism to escape local optima. In contrast, the GA algorithm showed a gradual increase in fitness value throughout the iterations, eventually converging at 0.88 with the selected subset FS4. However, it required 198 generations to reach convergence, making it the slowest in terms of convergence speed. In comparison, MPGH-FS achieved a higher search efficiency during evolution. Its fitness value increased rapidly by approximately 0.23 within the first 60 generations, reaching 0.88 by the 60th iteration, and then steadily converged to 0.89, corresponding to the final selected feature subset FS6. This process highlighted the strong global search capability and convergence efficiency of MPGH-FS. By effectively avoiding entrapment in local optima, the algorithm enhanced overall optimization performance, ultimately achieving significantly better accuracy and stability than both GA and HC.

#### 4.2.3. Performance Comparison and Analysis of Feature Selection Algorithms

The feature subsets obtained from the six feature selection methods are shown in Table 3. FS1 contained all 232 initial features, serving as the baseline set for comparison to evaluate the effectiveness of other feature selection methods in terms of dimensionality reduction and model performance improvement. In contrast, FS2 comprised 132 selected features. Statistical analysis revealed that, in addition to spectral, geometric, and index features, FS2 still retained 108 GLCM texture features, accounting for 82% of the subset. This suggested Relief-F + RFE retained a considerable degree of redundancy within the high-dimensional texture features. FS3 retained 47 features, including 21 texture features covering seven different GLCM attributes. Compared with FS2, this subset achieved a substantial reduction in texture dimensions, but nearly all spectral and geometric features were preserved, with no evident elimination. Compared with these two methods, the feature subsets derived from optimization algorithms demonstrated a more significant capability for dimensionality reduction. FS4 was primarily composed of vegetation indices (NGBDI, MGRVI) and spectral features (Brightness), reflecting GA’s advantage in identifying discriminative feature combinations. FS5, in contrast, was more focused on edge features (Border length) and local contrast (GLCM Contrast Layer 4), highlighting the local search characteristic of HC. Despite differing feature selection preferences, both subsets achieved a dimensionality reduction rate of 97.0%. The final feature subset selected by the MPGH-FS method, FS6, contained 9 features. This subset included key texture features such as GLCM Dissimilarity Layer 3 (0°) and StdDev (90°), vegetation indices like NDVI and NGRDI, as well as HSI saturation and spectral mean. This feature combination achieved a better balance between global discriminative power and local sensitivity.

Table 4 summarizes the samples’ classification accuracy results obtained using the feature subsets selected by the six methods, comparing their performance across four key metrics: number of selected features, fitness value, classification accuracy, and runtime. In terms of feature reduction, the Relief-F + RFE method reduced the original 232 features to 132, achieving a 43% reduction in dimensionality and improving sample accuracy to 90.12%. The RF + RFE method performed even better, eliminating about 80% of noisy features and achieving a classification accuracy of 92.83%. Although both methods are highly efficient, the retained features remained relatively redundant. In contrast, the three randomized optimization-based algorithms reduced the number of features to fewer than 10, demonstrating stronger dimensionality reduction capabilities. Among them, MPGH-FS selected an average of only 8.9 features, significantly reducing redundant features while maintaining classification performance. Regarding fitness values, MPGH-FS achieved an average fitness value of 0.89, significantly higher than those of GA and HC. This indicated a more favorable trade-off between classification accuracy and subset compactness, as well as superior convergence and search efficiency. When considering samples’ classification accuracy, MPGH-FS achieved an average accuracy of 94.92%, outperforming GA and HC by 3.08% and 9.00%, respectively. Additionally, its standard deviation was only 0.92%, notably lower than those of the other algorithms, indicating higher stability and generalization ability. From the perspective of computational efficiency, MPGH-FS had an average runtime of 23 min, which was slightly longer than HC but significantly shorter than GA’s 112 min, demonstrating its practical value.

Overall, MPGH-FS exhibited comprehensive advantages in feature compression, fitness trade-off, sample accuracy, and runtime, confirming its potential for high-dimensional feature selection tasks in high-resolution remote sensing imagery.

### 4.3. Full-Image Classification Accuracy Evaluation

**Figure 9:**
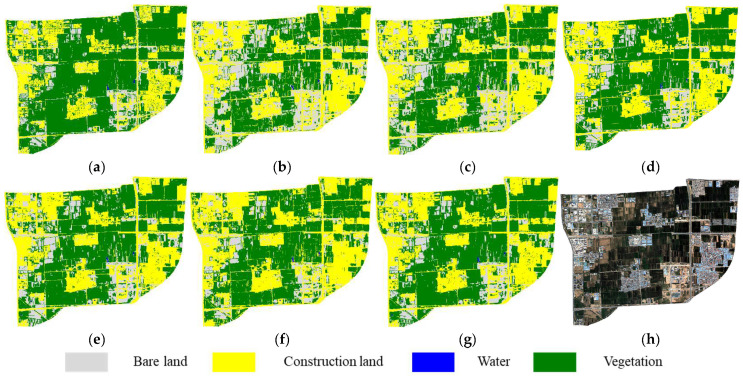
Full-scene classification results using different feature subsets: (**a**) Reference map. (**b**–**g**) Results using FS1–FS6 feature subsets. (**h**) Original imagery.

Based on the validation of sample-level classification performance, the six feature subsets (FS1–FS6) were further applied to classify the entire 2020 GF-2 image of the study area, enabling a visual comparison of the spatial performance of each algorithm. The results are shown in Figure 9. A systematic analysis of the classification results based on the six feature subsets revealed different feature selection methods had a significant impact on land cover classification. In particular, Figure 9b,c exhibited evident confusion between vegetation and bare land, especially in the lower right region of the image, where fragmented vegetation patches were misclassified as continuous bare areas. In contrast, the classification results using FS3–FS6 (Figure 9d–g) showed improved spatial consistency of land cover distribution. However, issues such as boundary expansion of construction areas and transitional zones around building and road persisted, hindering the accurate identification of small vegetated patches. Among them, the feature subset FS5 generated by the HC (Figure 9f) displayed the most prominent boundary blurring. By comparison, the proposed MPGH-FS method demonstrated relatively clear boundaries and more accurate recognition of fragmented vegetation, confirming the effectiveness of the multi-stage feature selection strategy (Figure 9g).

To comprehensively quantify and compare the performance of different feature selection methods in full-image classification, Overall Accuracy (OA) and the Kappa Coefficient were employed as evaluation metrics in this study. Meanwhile, the training and inference time of the SVM classifier using each feature subset was also recorded, as shown in Table 5. Compared to FS1, FS2 reduced the feature dimension from 232 to 132 through the Relief-F + RFE strategy, retaining about 57% of the total features. This dimensionality reduction led to a moderate improvement in classification accuracy, with OA increasing from 71.29% to 77.33% and Kappa rising from 0.55 to 0.63. However, its impact on computational efficiency was limited, with training and inference times reduced by only about 21%, indicating the persistence of considerable feature redundancy. FS3 further compressed the feature number to 47, accounting for about 20% of the total features, and achieved a notable improvement in classification accuracy, with OA reaching 84.37%, second only to the method proposed in this study. Nevertheless, its total training and Inference time still reached approximately 25 min.

In contrast, the three feature subsets FS4–FS6, derived from random optimization-based feature selection methods, compressed the feature space to approximately 4% of its original size, thereby substantially simplifying the classification models. As a result, both training and inference times decreased by over 97% compared to FS1, indicating a remarkable improvement in computational efficiency. Moreover, these subsets achieved notable improvements in classification accuracy. Among them, FS6 outperformed the others, achieving an OA of 85.55% and a Kappa coefficient of 0.75, with training completed in just 5.9 s and inference in around one minute. These results demonstrated that the proposed MPGH-FS method achieved a desirable balance between classification accuracy and computational efficiency, confirming its effectiveness for remote sensing image classification.

To further investigate the classification performance of each feature subset across different land cover categories, Table 6 presented the Producer’s Accuracy (PA) for each class. The PA for bare land was generally low, primarily because a substantial portion of the study area consists of construction sites. Although these surfaces appeared bare, they were often interspersed with various construction materials, soil piles, or debris, resulting in complex and unstable spectral and texture characteristics, which posed challenges even for visual interpretation. In addition, the accuracy for water bodies showed unusually large fluctuations. Except for FS3 and FS6, the PA for water did not exceed 75%, with the lowest being 58.33% and the highest 72.51%, while FS6 reaches 90.64%. The main reason for this significant variability is that water bodies account for only about 9% of the total area in the study site. This meant that the number of water pixels is relatively small, so misclassification or omission of individual water objects can disproportionately affect the overall PA calculation for this class.

### 4.4. Transfer Testing and Generalization Validation

**Figure 10:**
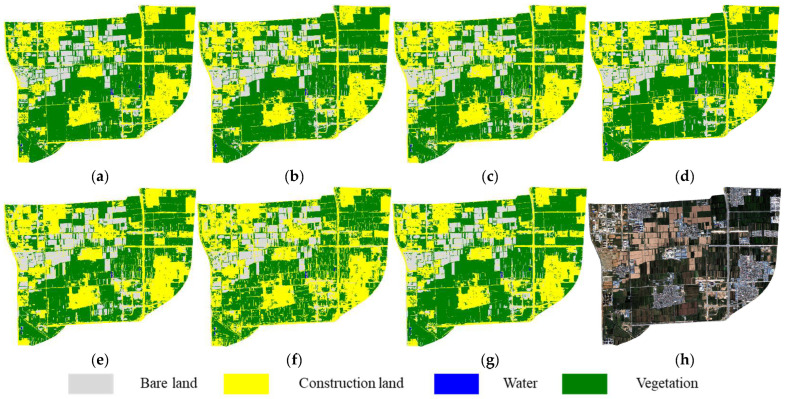
Cross-temporal transfer classification results (2018): (**a**) Reference map (**b**–**g**) Results using feature subsets FS1–FS6. (**h**) Original imagery.

**Figure 11:**
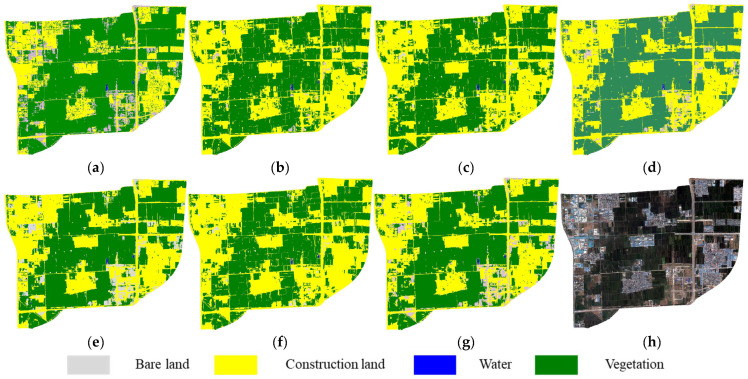
Cross-temporal transfer classification results (2019): (**a**) Reference map (**b**–**g**) Results using feature subsets FS1–FS6. (**h**) Original imagery.

**Figure 12:**
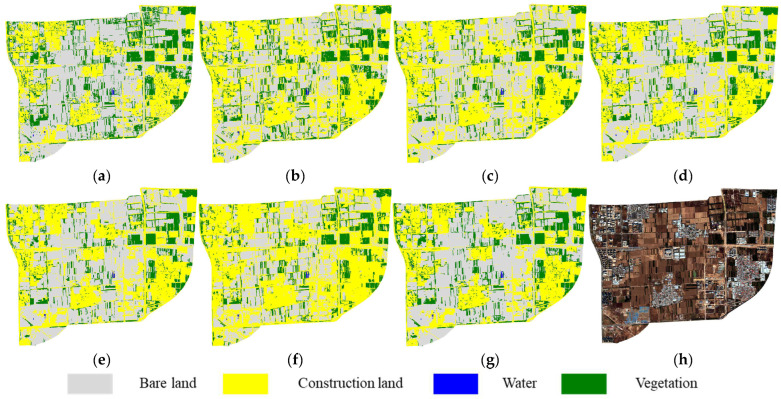
Cross-temporal transfer classification results (2022): (**a**) Reference map (**b**–**g**) Results using feature subsets FS1–FS6. (**h**) Original imagery.

**Figure 13:**
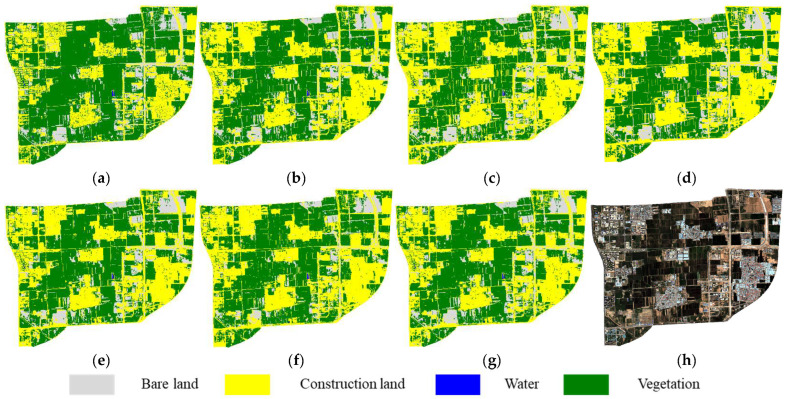
Cross-temporal transfer classification (2023): (**a**) Reference map for 2023. (**b**–**g**) Results using FS1–FS6 feature subsets. (**h**) Original imagery.

To further evaluate the temporal generalization capability of the proposed feature selection method, a cross-temporal transfer experiment was conducted. Specifically, the feature subsets (FS1–FS6), extracted from the July 2020 imagery using different methods, were directly applied to classify high-resolution remote sensing images of the same study area acquired in September 2018, July 2019, October 2022, and July 2023. To ensure a fair comparison, the feature subsets, classifier type, and parameter settings were kept consistent across all experiments, thereby enabling a more scientific evaluation of each method’s adaptability and robustness under cross-temporal conditions. The classification results are shown in Figure 10, Figure 11, Figure 12 and Figure 13.

In the September 2018 imagery, the presence of large, harvested farmland areas in the upper region of the study area resulted in distinct object boundaries and gradual transitions between land cover types. Moreover, large-scale urban development had not yet commenced, leading to relatively stable land cover distributions, which facilitated the transferability of the classification models. Consequently, most feature subsets yielded satisfactory classification performance on this imagery. Notably, FS5 exhibited visible construction land artifacts within vegetated areas (Figure 10f), while the results from the other subsets were largely consistent with the reference data. Among them, FS6 produced the most coherent classification map, with minimal noise and the closest spatial resemblance to the ground truth.

Vegetation cover in the study area was significantly greater in the July 2019 imagery (summer) compared to the July 2018 imagery. At the same time, a prominent construction site appeared on the left side, and patchy bare land caused by human activities emerged in the lower right corner. These changes led to some confusion between construction land and bare land in the classification results for most feature subsets. However, since the area of bare land was limited, the overall classification results still largely agreed with the reference map. Comparatively, the classification result using FS6 (Figure 11g) exhibited the least confusion.

The October 2022 imagery exhibited the largest seasonal difference compared to the other images, with extensive areas of bare land showing varying post-harvest shades and a significant reduction in vegetation cover, posing a considerable challenge to the adaptability of the feature subsets. In the classification results, FS5 (Figure 12f) showed a clear over-expansion of construction land, achieving the poorest agreement with the reference map. Considering the results from the previous two images, this phenomenon may indicate that the features selected in FS5 are insufficiently sensitive to variations in bare land color and texture, making it difficult to distinguish brighter bare land from actual construction land, especially under complex post-harvest surface conditions. In contrast, the feature subset selected in FS6 continued to demonstrate strong discriminative capability, yielding classification results that were closest to the reference map.

In the 2023 imagery, the study area experienced significant anthropogenic disturbances, including newly constructed roads in the right-hand region and extensive new construction land in the lower-left corner. These changes substantially increased the complexity of the land surface patterns, posing greater challenges for classification. Compared with the 2018 imagery, the overall classification accuracy of each feature subset declined slightly, with over-classification of construction areas commonly observed. The classification result using FS2 (Figure 13c) exhibited a high degree of fragmentation, especially in the transitional zones between cropland and construction land, where notable misclassifications occurred. In contrast, FS6 (Figure 13g) maintained superior classification performance, characterized by coherent object boundaries and minimal misclassifications. Its classification result remained highly consistent with the reference map, further validating the robustness and generalization capability of the feature subset selected by MPGH-FS across different temporal scales.

Based on the classification results obtained from the cross-temporal transfer experiments, this study quantitatively evaluated the temporal generalization ability of six feature subsets (FS1–FS6) using OA and Kappa coefficient. The classification performance of the six feature subsets across a total of five images was summarized in Table 7. Among them, FS6 performed best, achieving the highest classification accuracy in four out of five images, except for the 2018 imagery, with an average OA of 86.21%, which is 6.58% higher than the full feature set FS1. FS3 and FS4 showed similar performance, both with an average OA of 84%, indicating that the three feature selection strategies—MPGH-FS, RF + RFE, and standard GA—possess a certain degree of transferability over time, meeting the basic accuracy requirements for cross-temporal remote sensing classification. FS2 and the full feature set FS1 exhibited similar classification performance, with an average OA of around 80%, performing poorly on the 2020 and 2022 imagery. This may be partly due to the relatively close number of features they retain and also suggests that these two images’ classification tasks rely more heavily on effective feature dimensionality reduction. Among the feature selection methods, FS5 showed the most pronounced accuracy fluctuations, with the lowest OA reaching only 68.10% and the average OA even lower than FS1. This result is likely because the standard HC algorithm relies entirely on a random process to generate new feature selection results, lacking the global search guidance present in GA, which leads to poorer temporal generalization stability of the selected features.

A comprehensive analysis of the cross-temporal transfer experiments using five images clearly demonstrated the superior performance of FS6. It consistently achieved the highest OA and Kappa values among all subsets, while maintaining an exceptionally high feature compression ratio. This optimal balance between classification accuracy and dimensionality reduction substantially enhances computational efficiency without sacrificing performance. FS3 and FS4 exhibit slightly lower overall classification performance, with FS3 achieving less feature compression than FS6 and FS4 showing lower algorithm runtime efficiency than FS6. These results confirm the method’s strong generalization capacity and highlight its practical applicability in real-world cross-temporal remote sensing tasks, particularly in scenarios demanding high classification efficiency and low computational overhead.

## 5. Conclusions and Discussion

The MPGH-FS framework advances high-dimensional feature selection in OBIA through synergistic integration of MICC pre-filtering, GA global search, and HC local optimization. This three-stage architecture optimally balances selection efficiency and classification performance while addressing feature redundancy, model complexity, and cross-temporal adaptation challenges in high-resolution remote sensing.

Experimental results demonstrated MPGH-FS’s threefold advantage in multi-temporal GF-2 image classification. First, the framework achieved exceptional dimensionality reduction, compressing the feature set from 232 to 9 dimensions while retaining discriminative information. Second, the refined feature subset enables superior classification performance, with the highest overall accuracy (85.55%) and Kappa coefficient (0.75) on the 2020 image. Most notably, the method exhibited remarkable temporal consistency, with accuracy deviations remaining below 4% across the 2018–2023 test periods. These collective results position MPGH-FS as an efficient and reliable solution for operational time-series OBIA applications. These encouraging results can be attributed to the underlying mechanism of MPGH-FS. Specifically, its performance advantages primarily stem from the reasonable design of the multi-stage collaborative optimization strategy. The first stage, MICC pre-filtering, effectively eliminated a large number of redundant features, significantly reducing the search space. The second stage, GA, achieved global search and diversification of candidate feature subsets, enhancing the exploration capability of the solution space. The third stage, HC, refined and adjusted the subsets locally, improving the overall quality of the final feature subset. The complementary combination of these three stages not only accelerated the optimization convergence speed but also effectively avoided local optima, demonstrating strong model stability and versatility.

Although this method demonstrated strong performance in both feature selection efficiency and classification accuracy, several limitations remain. First, the stability of the selected features remained affected by the quality of the original feature extraction, particularly in scenarios characterized by high target heterogeneity or substantial image noise, where the robustness of the method still requires improvement. Second, the current experimental validation relied solely on five temporal GF-2 images and was not extended to more complex temporal patterns or multi-source remote sensing datasets. Third, the data preprocessing steps could be further improved; for example, image denoising could be performed using the Panchromatic Weighted Representation Coefficient Total Variation (PWRCTV) method to mitigate noise caused by image artifacts, thereby further enhancing feature selection and image classification performance [39]. Future research should further explore the method’s adaptability across broader spatial–temporal ranges, diverse geographic regions, and heterogeneous data sources to improve its generalizability and practical utility.

## Figures and Tables

**Table 1:** Extracted feature categories and descriptions.

Type	Name	Count	Description
Spectral Features	Brightness, Standard deviation Layer 1, Standard deviation Layer 2, Standard deviation Layer 3, Standard deviation Layer 4, Max. diff., Mean Layer 1, Mean Layer 2, Mean Layer 3, Mean Layer 4, HSI Transformation Hue, Intensity, Saturation	13	Object brightness, mean pixel values, standard deviation, etc.
Geometric Features	Area, Area (excluding inner polygons), Area (including inner polygons), Border length, Length, Length/Width, Number of pixels, Width	8	Object area, length, width, and perimeter, etc.
Index Features	ExB, ExG, ExR, MGRVI, NDVI, NGBDI, NGRDI, NRI, RGBVI, VARI, VDVI	11	Visible light indices and vegetation indices
Texture Features	GLCM Ang. 2nd moment, GLCM Contrast, GLCM Correlation, GLCM Dissimilarity, GLCM Entropy, GLCM Homogeneity, GLCM Mean, GLCM StdDev	200	Gray-Level Co-occurrence Matrix (GLCM) with 8 attributes

**Table 2:** Comparison of Feature Selection Methods.

Method	Category	Selected Feature Subset	Description
No Feature Selection	Baseline	Feature Subset 1 (FS1)	Retains the original 232 features
Relief-F + RFE	Statistical Method	Feature Subset 2 (FS2)	Combines statistical evaluation with recursive feature elimination
RF + RFE	Machine Learning Method	Feature Subset 3 (FS3)	Combines machine learning with recursive feature elimination
GA	Randomized Optimization Method	Feature Subset 4 (FS4)	Global search-based feature selection
HC		Feature Subset 5 (FS5)	Greedy local search optimization
MPGH-FS		Feature Subset 6 (FS6)	Hybrid intelligent optimization

**Table 3:** Composition of feature subsets generated by five different selection methods.

Feature Subset	Content	Count
FS1	All of the features	232
FS2	ExB, ExG, ExR, HSI Transformation Hue, Intensity, Saturation, Length/Width, Max. diff., Mean Layer 1, 2, 3, 4, MGRVI, NDVI, NGBDI, NGRDI, NRI, RGBVI, Standard deviation Layer 1, 2, 3, 4, VARI, VDVI, GLCM Ang. 2nd moment, Contrast, Correlation, Dissimilarity, Entropy, Homogeneity, Mean, StdDev, etc.	132
FS3	Brightness, ExB, ExG, ExR, HSI Transformation Hue, Intensity, Saturation, Length, Length/Width, Max. diff., Mean Layer 1, 2, 3, 4, MGRVI, NDVI, NGBDI, NGRDI, NRI, RGBVI, Standard deviation Layer 1, 2, 3, 4, VARI, VDVI, GLCM Ang. 2nd moment, Contrast, Correlation, Dissimilarity, Homogeneity, Mean, StdDev	47
FS4	NGBDI, MGRVI, Max. diff., Length, GLCM Correlation Layer 1 (all dir.), Standard deviation Layer 2, Brightness.	7
FS5	Border length, ExR, GLCM Contrast Layer 4 (90°), GLCM Contrast Layer 4 (0°), Standard deviation Layer 4, Mean Layer 1, 4.	7
FS6	GLCM Dissimilarity Layer 3 (0°), GLCM StdDev (90°), Standard deviation Layer 3, HSI Transformation Saturation, NDVI, Max. diff., Mean Layer 2, NGRDI, ExB.	9

**Table 4:** Performance comparison of five feature selection methods on sample classification tasks.

Method	Fitness	Feature Count	Sample Accuracy	Runtime
All features	—	232	86.11%	—
Relief-F + RFE	—	132	90.12%	<1 min
RF + RFE	—	47	92.83%	3 min
GA	0.85 ± 0.02	9.5 ± 1.8	91.84% ± 1.31%	112 min ± 11 min
HC	0.81 ± 0.01	6.9 ± 0.9	85.92% ± 1.55%	17 min ± 1 min
MPGH-FS	0.89 ± 0.01	8.9 ± 1.8	94.92% ± 0.92%	23 min ± 1 min

**Table 5:** Comparison of full-scene classification accuracy and computational time across FS1–FS6.

	FS1	FS2	FS3	FS4	FS5	FS6
OA	71.29%	77.33%	84.37%	83.94%	80.44%	85.55%
Kappa	0.55	0.63	0.73	0.73	0.67	0.75
Train Time	6 min	3 min	1 min 5 s	1.9 s	1.5 s	5.9 s
Inference Time	83 min	67 min	23 min 48 s	25.7 s	20.3 s	1 min 14 s

**Table 6:** Classification accuracies of different feature subsets (FS1–FS6) across land cover classes.

Class	Feature Subset					
	FS1	FS2	FS3	FS4	FS5	FS6
Bare land (%)	63.97	69.76	69.23	71.08	61.06	65.24
Vegetation (%)	66.15	74.42	83.32	82.1	79.85	85.57
Water (%)	58.33	59.26	86.49	72.51	64.14	90.64
Construction land (%)	87.80	88.61	72.03	95.69	92.87	97.06

**Table 7:** Classification performance of six feature subsets across five temporal GF-2 images.

		FS1	FS2	FS3	FS4	FS5	FS6
2018	OA	87.55%	86.80%	89.64%	87.70%	79.84%	89.39%
	Kappa	0.79	0.78	0.82	0.8	0.66	0.82
2019	OA	83.17%	83.18%	84.91%	85.20%	79.15%	87.15%
	Kappa	0.68	0.68	0.72	0.73	0.62	0.76
2020	OA	71.29%	77.33%	84.37%	83.94%	80.44%	85.55%
	Kappa	0.55	0.63	0.73	0.73	0.67	0.75
2022	OA	72.09%	75.74%	80.77%	81.17%	68.10%	84.47%
	Kappa	0.56	0.61	0.69	0.69	0.51	0.75
2023	OA	84.04%	78.56%	81.61%	83.64%	81.93%	84.48%
	Kappa	0.72	0.64	0.69	0.72	0.69	0.74
Average	OA	79.63%	80.32%	84.26%	84.33%	77.89%	86.21%
	Kappa	0.66	0.67	0.73	0.73	0.63	0.76

## Data Availability

The original contributions presented in the study are included in the article, further inquiries can be directed to the corresponding author.
